# Fatigue in fluctuating Parkinson’s disease patients: possible impact of safinamide

**DOI:** 10.1007/s00702-023-02654-1

**Published:** 2023-05-20

**Authors:** Caterina Pauletti, Nicoletta Locuratolo, Daniela Mannarelli, Andrea Maffucci, Alessia Petritis, Elisa Menini, Francesco Fattapposta

**Affiliations:** 1 Department of Human Neurosciences, Universita degli Studi di Roma La Sapienza, Rome, Italy; 2 National Centre for Disease Prevention and Health Promotion, National Institute of Health, Rome, Italy; 3 Department of Neurosciences, Biomedicine and Movement Sciences, University of Verona, Verona, Italy

**Keywords:** Non-motor symptoms, Parkinson’s disease, Fatigue, Glutamate, Safinamide, Neuroinflammation

## Abstract

Fatigue is a common non-motor symptom in Parkinson’s disease (PD). Among other pathophysiological mechanisms, neuroinflammation, a pathological PD hallmark associated with changes in glutamatergic transmission in basal ganglia, has been proposed as a crucial factor closely related to fatigue. To test the hypothesis that safinamide could represent an effective treatment of fatigue in PD patients, given its dual mechanism of action (it selectively and reversibly inhibits MAOB and modulates glutamate release), we administered the validated versions of fatigue severity scale (FSS) and Parkinson fatigue scale-16 (PFS-16) to 39 fluctuating PD patients with fatigue before and after a 24-week treatment period with safinamide as add-on therapy. An assessment of secondary variables such as depression, quality of life (QoL), and motor and non-motor symptoms (NMS) was conducted. After 24 weeks of treatment with safinamide, both FSS (*p* < 0.001) and PF-S16 (*p* = 0.02) scores were significantly lower than at baseline. Moreover, 46.2% and 41% of patients scored below the cut-off for the presence of fatigue according to FSS and PFS-16, respectively (responders). At follow-up, a significant difference emerged between responders and non-responders in mood, QoL, and NMS. Fatigue improved in fluctuating PD, and more than 40% of patients were “fatigue-free” after a 6 month treatment with safinamide. Patients without fatigue at follow-up displayed significantly better scores in QoL domains, such as mobility or activities of daily living, although disease severity remained stable, supporting the hypothesis that fatigue could considerably affect QoL. Drugs that interact with multiple neurotransmission systems, such as safinamide, could be useful in reducing this symptom.

## Introduction

Fatigue, defined as a subjecting feeling of an overwhelming lack of energy or a need for increased effort during daily activities (Siciliano et al. 2022), is a common non-motor symptom of PD (Friedman et al. 2007).

Up to 58% of the PD population experience fatigue (Stocchi et al. 2014), which may emerge at the early stages of the disease, even in the prodromal phase, and it tends to remain stable during the disease progression (Schifitto et al. 2008; Barone et al. 2009; Siciliano et al. 2020). Even if fatigue is often associated with anxiety, depression, and sleep disorders, it is an independent symptom, being present also in non-depressed, non-demented, and non-sleepy PD patients (Shulman et al. 2001; Friedman et al. 2007). Besides it being common, fatigue in PD is also a distressing and disabling symptom, leading to limitations in patients’ quality of life (Barone et al. 2009; Herlofson and Kluger 2017). Moreover, it represents one of the most common non-motor fluctuations (Witjas et al. 2002; Storch et al. 2013).

Fatigue in PD is characterized by a sense of exhaustion disproportionate to attempted activities, often chronic and, as aforementioned, pathological, i.e., clinically significant, associated with nonrestorative rest, and involving both motor and cognitive domains (Friedman et al. 2011; Kluger et al. 2013; Kluger 2017).

Despite its relevance, its pathophysiological mechanisms are still not fully understood. So far, a dysfunction of the striato-thalamo-cortical loop connecting the neostriatum to the prefrontal cortex and responsible for processing the costs of acting (Boksem and Tops 2008) has been hypothesized (Chaudhuri and Behan 2000; Pauletti et al. 2019).

Even if dopamine loss is crucial in the development of PD clinical picture, much evidence demonstrated that dopaminergic pathways are only partially involved in the pathogenesis of fatigue and other mechanisms have to be taken into account: no association between nigrostriatal dopaminergic degeneration and fatigue has been demonstrated so far (Schifitto et al. 2008; Pavese et al. 2010; Kang et al. 2020); moreover, the association between fatigue and motor symptoms and its response to dopaminergic treatment are inconsistent (Schifitto et al. 2008; Siciliano et al. 2018; Lazcano-Ocampo et al. 2020). Evidence supports the role that disruption of non-dopaminergic pathways, especially serotonergic pathways, possibly leading to a serotonin/dopamine imbalance in the striatum, might be involved in the pathophysiology of fatigue (Pavese et al. 2010; Siciliano et al. 2018; Pauletti et al. 2021), but data are still inconclusive.

In recent years, much evidence suggested that glutamatergic overactivity, linked to neurodegeneration and responsible for excitotoxicity, is crucial in inducing several striatal alterations in early and advanced symptomatic stages of PD (Ambrosi et al. 2014; Campanelli et al. 2022), and may be related to the motor as well as non-motor symptoms, such pain, cognitive impairment, mood, and sleep disorders (Barone 2010; Geroin et al. 2020; Rinaldi et al. 2021). Glutamatergic overactivity, as well as excitotoxicity, are now well-known mechanisms crucially contributing to the promotion of neuroinflammation in PD (Iovino et al. 2020; Wang et al. 2020), which has been recently associated with the presence of primary fatigue (Morris et al. 2015, 2016).

Safinamide is an orally administered alfa-aminoamide derivative, currently used in the treatment of fluctuating PD, given its dual mechanism of action: it selectively and reversibly inhibits monoamine oxidase-B (MAOB), and modulates glutamate release: it reduces subthalamic/nigral glutamatergic hyperactivity through use-dependent sodium channel blockade, which prevents calcium channel opening (Pagonabarraga et al. 2021; Stocchi et al. 2022).

The aim of the present study was to test the hypothesis that safinamide, i.e., a drug that targets both dopaminergic and glutamatergic systems could represent an effective treatment of fatigue in PD patients, given its dual mechanism of action.

We hypothesized that if fatigue is, at least partially, related to increased levels of extracellular glutamate, using safinamide in fluctuating PD patients will ameliorate this symptom.

## Methods

### Design

We used an observational, prospective, single-center pilot study of 24 weeks to evaluate the effects of safinamide on fatigue in patients with idiopathic PD in the mid-late stage of the disease, suffering from motor fluctuation. The study was carried out at the Department of Human Neuroscience, Sapienza University of Rome. The study was conducted following the Declaration of Helsinki and was approved by the local ethics committee. All the participants gave their written informed consent to the study.

### Subjects

Outpatients with a diagnosis of idiopathic PD in the mid-late stage of the disease, suffering from motor fluctuations, were recruited for the study. The presence of motor fluctuations was defined by the presence of > 1.5 h off time/day. Inclusion criteria were the following: > 18 years of age; confirmed diagnosis of idiopathic PD according to the UK Brain Bank diagnostic criteria (Gibb and Lees 1988); presence of motor fluctuations defined as > 1.5 h’ off time/day; stable optimized anti-parkinsonian therapy (for at least 4 weeks), always including levodopa; presence of fatigue, assessed according to definition criteria for PD-related fatigue on the basis of expert consensus (Kluger et al. 2016); the severity of fatigue was assessed by means of the Fatigue Severity Scale (FSS), which is a patient-rated brief and easy unidimensional 9-item fatigue rating scale and the most frequently used fatigue-specific scale in chronic diseases (Krupp 1989) and using the 16-item Parkinson’s Fatigue Scale (PFS-16), which is a patient-rated scale that reflects the physical aspects of fatigue in patients with PD and measures both the presence of fatigue and its impact on daily function (Brown et al. 2005). Both scales have been recommended to assess the presence and severity of fatigue in PD populations (Friedman et al. 2010). Using the full Likert scale, an average score of FSS > 4 and PFS-16 ≥ 2.95 are used to distinguish patients who experienced fatigue from those who did not.

Exclusion criteria were the following: the presence of dementia (age- and education-adjusted MMSE score < 23.8, which is the cut-off for dementia according to Italian normative data (Measso et al. 1993)); major psychiatric comorbidities, including major depressive disorders and anxiety disorders; severe and progressive medical illnesses, including heart failure or OSAS; concomitant use of MAO inhibitors, tricyclic antidepressants, serotonin–norepinephrine reuptake inhibitors and beta-blockers; patients for whom safinamide is contraindicated.

### Assessment

At the enrollment (T0), all patients were examined by two independent, experienced neurologists. The severity of PD was assessed by the Hoehn and Yahr staging scale, while motor disability was rated employing the Unified Parkinson’s Disease Rating Scale-subset III (UPDRS III), with both being assessed during the on phase. Moreover, validated questionnaires were used to assess depression (Beck depression inventory—BDI (Beck 1961)), quality of life (39-item Parkinson’s Disease Questionnaire—PDQ-39) (Peto et al. 1995), motor and non-motor fluctuations (wearing-off questionnaire WOQ (Abbruzzese et al. 2012)), and non-motor symptoms (non-motor symptom scale—NMSS (Chaudhuri et al. 2007)). In particular, given the association between fatigue and other non-motor symptoms such as apathy, daytime somnolence and sleep disorders (Skorvanek et al. 2013), above total NMSS and its 9 domains, the NMSS items related to Sleep (domain 2—sleep/fatigue, item 3: daytime sleepiness; item 5: difficulties falling or staying asleep; item 6: restless legs) and to apathy (domain 3—mood/cognition, item 7: lost interest in surroundings; item 8: lack of motivation; item 11: flat mood) (Chaudhuri et al. 2007, 2013) were also individually assessed and reported.

Safinamide was started at 50 mg/day (once daily) for 15 days, then up-titrated to 100 mg/day. During the observational study period, the dosage of other dopaminergic drugs was kept stable unless the neurologist deemed changes indispensable.

After 12 weeks (T1), a clinical assessment was performed, and side effects were collected. After 24 weeks (T2) of treatment, subjects underwent the clinical assessment and the evaluation of fatigue, and all the questionnaires above were administered.

### Statistical analysis

#### Sample size definition

Mean FSS score in the parkinsonian population ranges between 3.9 and 4.4; given the MCDI of FSS = 0.6 and based on alpha = 0.05 and a beta of 0.2 (power = 80%), a sample size of 37 patients was required to detect a clinical improvement due to safinamide using a Wilcoxon signed-rank test (one sample). (Calculated by G*Power). Considering a drop-out of 20%, 45 patients were enrolled.

**Analysis** Data are expressed as means (± standard deviation) for continuous variables and proportions for categorical variables. The Shapiro–Wilk test was used to assess the normal distribution of the data.

To verify the effect of safinamide on fatigue, a double approach was adopted. First, the Wilcoxon rank sign test was used to evaluate possible differences after treatment (baseline comparisons to 24 weeks) for FSS and PFS-16 scores. Moreover, we calculated the proportion of responders as indicated by the proportion of subjects obtaining at T2, a score in FSS < 4 and PFS-16 < 2.95, which indicated the absence of fatigue.

Wilcoxon rank sign test was used to evaluate possible differences after treatment for each other variable in the exam. Possible differences between responders and no-responders in secondary outcomes (disease severity, depression, quality of life, wearing-off, and non-motor symptoms) were tested using the Mann–Whitney test.

The Spearman rank correlation coefficients were performed to detect correlations between the clinical variables and fatigue.

A *p* < 0.05 will be considered as statistically significant. All the analyses were performed using the SPSS statistical package (version 27.0).

## Results

Forty-five outpatients were enrolled in the study. One patient dropped out because of the occurrence of a femoral fracture. Four patients needed a modification in the anti-parkinsonian therapy at T1, and one patient experienced hypotension and discontinued safinamide. The analyses were, therefore, conducted on 39 patients. The main clinical characteristics of the PD population are shown in Table 1.

**Table 1:** Clinical characteristics of PD population

	*n* = 39
Age (years)	68.2 ± 7.5 (48–80)
Sex (M/F)	26/13
Education (years)	10.7 ± 4.0 (4–18)
MMSE	28.9 ± 0.3 (24–30)
Age at onset	58.7 ± 1.3
Disease duration (years)	9.5 ± 4.9
UPDRS III	24.2 ± 8.4
HY (%)	
1	1 (2.6%)
2	19 (48.7%)
2.5	7 (17.9%)
3	12 (30.8%)
Predominant motor symptom (%)	
Tremor	10 (25.6%)
Rigidity	19 (48.7%)
Mixed	10 (25.6%)
LEDD (mg/die)	842.5 ± 354.0 (300–1510)
Familiarity (y/n)	7/32

Data are expressed as mean ± standard deviation (median; range)

*MMSE* mini mental state examination; *UPDRS III* unified Parkinson’s disease rating scale part III; *HY* Hohen–Yahr stage; *LEDD* levodopa equivalent daily dose

A significant improvement in fatigue was observed after 24 weeks of treatment with safinamide. Both FSS (pre: 5.1 ± 1.4, post: 4.2 ± 1.6; *p* < 0.001) and PFS-16 (pre:3.5 ± 0.9, post:3.2 ± 0.9; *p* = 0.02) showed a significant reduction. Moreover, 18/39 (46.2%) and 16/39 (41%) obtained scores, respectively, at FSS and PFS-16, below the cut-off for the presence of fatigue.

No significant difference emerged in BDI, PDSI, WOQ-19, and total NMSS, sleep-related NMSS items, apathy-related NMSS items. Results are displayed in Table 2.

**Table 2:** Changes in scores of assessment scales after 6 months of treatment with safinamide

(*n* = 39)	Baseline Mean ± SD	Follow up Mean ± SD	*P*
UPDRS III—on	24.2 ± 8.4	25.1 ± 9.2	0.34
FSS	5.1 ± 1.4	4.2 ± 1.6	** < 0.001**
PFS-16	3.5 ± 0.9	3.2 ± 0.9	**0.02**
BDI	12.0 ± 8.3	10.5 ± 7.7	0.26
PDQ-39 (PDSI)	32.3 ± 13.5	30.2 ± 11.5	0.45
WOQ-19	6.5 ± 4.2	7.0 ± 4.0	0.39
Total NMSS	68.7 ± 37.1	77.6 ± 48.1	0.28
NMSS—Sleep			
Daytime sleep	1.1 ± 1.9	2.4 ± 3.8	0.08
Difficulties falling or staying asleep	4.0 ± 4.5	4.0 ± 4.5	0.75
Restless legs	2.9 ± 3.6	3.2 ± 3.6	0.79
NMSS—apathy			
Lost interest in surroundings	1.4 ± 2.9	1.5 ± 3.2	0.81
Lack motivation	1.7 ± 3.2	2.8 ± 3.9	0.10
Flat mood	0.5 ± 1.1	0.8 ± 2.2	0.57

Data are expressed as mean ± standard deviation. Wilcoxon rank sign test. Significant values (*p* < 0.05) are highlighted in bold

*UPDRS III* unified Parkinson’s disease rating scale part III. *FSS* fatigue severity scale; *PFS-16* Parkinson’s disease fatigue scale; *BDI* Beck depression inventory; *WOQ* wearing-off questionnaire; *PDQ-39* Parkinson’s disease questionnaire; *PDSI* Parkinson’s disease summary index; *NMSS* non-motor symptoms scale

After splitting the PD population at T2 accordingly to the presence of fatigue (FSS < 4 PD patients without fatigue “responders”; FSS > 4 PD patients with fatigue “non-responders”), a significant difference between groups emerged in BDI, PDSI, and NMSS. No difference emerged between groups in demographic or clinical characteristics, and between the variables at T0 mentioned above. Results are displayed in Table 3.

**Table 3:** Differences between responders (PDnF) and non-responders (PDF) at follow-up

(*n* = 39)	PDnF at T2 (“responders”)	PDF at T2 (“non-responders”)	*P*
*n*	18 (46.2%)	21 (53.8%)	
Age (years)	66.9 ± 7.8	69.4 ± 7.1	0.39
Sex (M/F)	12/6	14/7	0.63°
Age at onset	59.2 ± 8.0	58.3 ± 8.0	0.77
Disease duration (yrs)	8.0 ± 3.2	10.8 ± 5.8	0.06
LEDD (mg/die)	786.9 ± 394.2	892.0 ± 315.4	0.26
FSS	2.9 ± 0.8	5.4 ± 1.0	** < 0.001**
UPDRS III—on	22.7 ± 7.9	27.1 ± 9.9	0.24
BDI	8.7 ± 8.0	12.3 ± 7.2	**0.05**
WOQ-19	6.9 ± 4.0	7.1 ± 4.1	0.93
PDQ-39 (PDSI)	26.5 ± 12.1	33.8 ± 9.9	**0.05**
Mobility	13.8 ± 9.3	22.1 ± 5.6	**0.005**
Activities of daily living	6.7 ± 4.3	10.8 ± 5.7	**0.02**
Emotional well-being	8.9 ± 5.5	10.5 ± 4.7	0.32
Stigma	3.1 ± 3.3	3.4 ± 3.4	0.96
Social support	1.2 ± 1.8	0.9 ± 1.7	0.83
Cognitions	3.4 ± 3.2	4.8 ± 2.7	0.09
Communication	3.4 ± 3.1	2.9 ± 0.5	0.94
Bodily discomfort	4.1 ± 3.1	5.1 ± 2.7	0.31
NMSS tot	52.9 ± 34.0	100.9 ± 48.5	**0.001**
Cardiovascular	0.8 ± 2.0	3.1 ± 3.6	**0.01**
Sleep/fatigue	10.9 ± 8.4	17.7 ± 10.4	**0.05**
Mood/apathy	6.9 ± 9.1	17.1 ± 14.1	**0.009**
Perceptual problems/hallucinations	2.5 ± 4.5	1.9 ± 4.3	0.56
Attention/memory	3.8 ± 7.3	7.6 ± 10.0	0.10
Gastrointestinal tract	8.5 ± 8.3	11.2 ± 8.7	0.31
Urinary symptoms	10.2 ± 7.2	21.4 ± 12.6	0.07
Sexual function	2.7 ± 4.7	10.1 ± 8.8	**0.009**
Miscellaneous	6.6 ± 6.9	10.8 ± 2.3	0.22

Data are expressed as mean ± standard deviation (median; range). Mann–Whitney *U* test; °chi-squared test. Significant values (*p* < 0.05) are highlighted in bold

*LEDD* Levodopa equivalent daily dose; *FSS* fatigue severity scale; *UPDRS III* unified Parkinson’s disease rating scale part III; *BDI* Beck depression inventory; *WOQ* wearing-off questionnaire; *PDQ-39* Parkinson’s disease questionnaire; *PDSI* Parkinson’s disease summary index; *NMSS* non-motor symptoms scale

No significant correlation emerged between fatigue and clinical characteristics of the PD population, such as age, age at onset, disease duration, disease severity-UPDRSIII. Moreover, fatigue severity at T0, did not correlate to BDI, WOQ-19, total NMSS or NMSS items related to sleep and apathy. A significant correlation emerged between fatigue and PDSI. Results are displayed in Table 4.

**Table 4:** Correlations between fatigue and clinical variables

(*n* = 39)	Rho	*p*
FSS—PFS-16	0.61	** < 0.001**
FSS—Age	0.29	0.08
FSS—age at onset	0.18	0.28
FSS—disease duration	0.16	0.33
FSS—UPDRS III	– 0.11	0.53
FSS—LEDD	– 0.10	0.56
FSS—BDI	0.23	0.15
FSS—WOQ-19	0.01	0.94
FSS—PDQ-39 (PDSI)	0.31	**0.05**
FSS—total NMSS	0.20	0.23
FSS—NMSS (Sleep)		
FSS—daytime sleepiness	0.14	0.40
FSS—falling/staying asleep	0.08	0.63
FSS—restless legs	0.01	0.94
FSS—NMSS (Apathy)		
FSS—lack of interest for surroundings	0.14	0.39
FSS—lack of motivation	0.08	0.64
FSS—flat mood	0.17	0.30

Spearman rank correlation coefficients. Significant values (*p* < 0.05) are highlighted in bold

*FSS* fatigue severity scale; *PFS-16* Parkinson’s disease fatigue scale; *LEDD* levodopa equivalent daily dose; *UPDRS III* unified Parkinson’s disease rating scale part III; *BDI* Beck depression inventory; *WOQ* wearing-off questionnaire; *PDQ-39* Parkinson’s disease questionnaire; *PDSI* Parkinson’s disease summary index; *NMSS* non-motor symptoms scale

## Discussion

The present study aimed to evaluate the impact of safinamide, a drug that, besides its activity as a reversible MAOB inhibitor, at a higher dose (100 mg), inhibits glutamate release at overactive synapses by blocking voltage-dependent calcium and sodium channels (Alborghetti and Nicoletti 2019; Jost 2022), on PD-related fatigue.

After 6 months of treatment with safinamide at 100 mg/die, a significant reduction in fatigue scores was obtained. Moreover, more than 40% of our population obtained scores at fatigue-related scales, indicating fatigue’s absence.

Safinamide has been proved effective in improving motor functions and fluctuations in fluctuating PD patients, and more recently, many reports demonstrated its role also in reducing non-motor symptoms, such as mood fluctuations, sleep disturbances or chronic pain (Cattaneo et al. 2017; Geroin et al. 2020; Abbruzzese et al. 2021; Labandeira et al. 2021; Santos García et al. 2022).

The efficacy of safinamide on fatigue in fluctuating patients has been recently reported by few papers focusing on the effect of this drug on non-motor symptoms. De Micco et al. (2022) reported an effect of safinamide 50 mg on PFS-16 score on a cohort of 20 PD patients consecutively recruited to evaluate a possible improvement on non-motor, cognitive, and behavioral symptoms, in which the mean score of PFS-16 at baseline was slightly under the cut-off for the presence of fatigue (Friedman et al. 2010). Similarly, Santos García et al. (2021) reported an improvement in the NMSS-fatigue/sleep domain with safinamide. Our data are in line with these observations, and, to our knowledge, this is the first study to focus exclusively on patients that specifically suffered from PD-related fatigue, according to clinical scale scores and clinical criteria (Siciliano et al. 2022).

Even though PD-related fatigue is a well-known and common problem in the parkinsonian population, little is known about its pathophysiology.

There is evidence that elevated pro-inflammatory cytokines are intimately involved in the genesis and maintenance of the pathological levels of fatigue (Morris et al. 2016) and a contribution of dopaminergic and non-dopaminergic pathways have been demonstrated (Kluger et al. 2013; Kluger 2017). Some insight on the role of glutamatergic overactivity in fatigue comes from studies with patients with multiple sclerosis, a CNS demyelinating disease in which fatigue is extremely common and disabling, that demonstrated, using magnetic resonance spectroscopy, increased glutamine + glutamate/creatine ratios in frontal, parietal, and occipital lobes as well as in hypothalamic regions of patients with severe fatigue compared to a non-fatigued group or healthy subjects, glutamine/glutamate concentrations in the prefrontal cortex strongly correlated with total fatigue scores in MS patients. (Kantorová et al. 2017; Arm et al. 2021). As regards PD, there is growing evidence that neuroinflammation, a pathological hallmark of PD, plays a crucial factor in its genesis (Barnum and Tansey 2012; Wang et al. 2021). A high concentration of IL-6 has been demonstrated in the serum of PD patients with fatigue compared to patients without fatigue (Pereira et al. 2016); increased levels of inflammatory markers in cerebrospinal fluid were significantly associated with more severe symptoms of fatigue (Lindqvist et al. 2013) in this disease.

The interplay between inflammation and glutamatergic transmission is complex and goes far beyond its effect on glutamate reuptake. Although the observed effect of safinamide on fatigue in the present study is based only on clinical data, without any objective measurement of glutamatergic transmission, it may be considered that safinamide contributed to reducing fatigue targeting dopaminergic and non-dopaminergic systems, and that the reduction of glutamatergic overactivity may have played a role in our results.

In our sample, fatigue seems to be a symptom intrinsic to the pathological substrates associated with PD rather than secondary to the motor impairment: no correlation was found with clinical characteristics of the disease, such as disease severity, duration, and dopa replacement therapy. This data is in line with current literature: in most studies, the presence and severity of fatigue do not correlate with the duration or severity of disease, and a consistent correlation between the presence of fatigue and type, dosage, or duration of dopaminergic drug treatment has not been demonstrated (Friedman and Friedman 1993; Shulman et al. 2001; Stocchi et al. 2014; Siciliano et al. 2018). Fatigue is often associated to other non-motor symptoms such as sleep disturbances, depression, or apathy (Skorvanek et al. 2013), and although especially the latter two symptoms may be considered very close to fatigue, being characterized, similarly to fatigue, by a significant reduction in self-initiated voluntary actions (Kuppuswamy 2017), they are nowadays considered independent and distinct symptoms. Although we did not administer specific scales related to sleep problems and apathy in the present study, which represents a limitation of the study, the specific NMSS items dedicated to these non-motor symptoms, did not reveal significant changes at follow-up, and did not show any correlation to fatigue indicators such as FSS and PFS-16, indirectly indicating that these factors did not affect our results. It is possible that the fact that our sample was selected exclusively for the presence of primary fatigue, and that the severity of these non-motor symptoms was relatively low in our sample, prevented us from finding an effect of safinamide on sleep disturbance or apathy, as described in other reports (Santos García et al. 2021, 2022; De Micco et al. 2022).

When we analyzed subjects according to the proportion of responders, we observed that the two subgroups differed in many aspects at follow-up.

Even if patients overall displayed no or mild depression (as indicated by the mean score at BDI in both groups), PDnF (responders) displayed a significantly lower score at BDI than PDF (non-responders). The relationship between fatigue and depression is complex; these two symptoms are often associated in PD patients and fatigue, besides being one of the cardinal symptoms included in the diagnosis of depression, could be secondary to this feature (Kluger et al. 2016; Kluger 2017; Herlofson and Kluger 2017). However, in our study, psychiatric comorbidities, including major depression episodes, were considered as exclusion criteria to avoid the presence of secondary fatigue. Our results seem to indicate that an improvement in fatigue could lead to a better mood, or in alternative that patients that respond to safinamide, may benefit from this in BDI scores. Previous studies demonstrated the effectiveness of safinamide over mood (Peña et al. 2021a, b; Labandeira et al. 2021) and a role of glutamate overactivity on mood disorders has been hypothesized.

At the end of the follow-up, responder patients differed from non-responders in several quality-of-life outcomes: PDQ-39 global score (PDSI—summary index), mobility and ADL dimensions, and fatigue severity correlated with quality of life. A difference bordering on significance was found in the cognition dimension. This finding seems in line with previous literature indicating that fatigue is often associated with poor quality of life (Friedman et al. 2007; Barone et al. 2009). Interestingly, after treatment, patients without fatigue obtained better scores in mobility and activities of daily life, even if motor scores did not improve. This is not surprising, given the fact that fatigue is represented by a subjective sensation of tiredness, lack of energy, or exhaustion, which contributes directly and indirectly to restriction in activity and participation in daily activities, and could be defined as a failure in initiating and maintaining both attentional and physical tasks that require self- motivation (Friedman and Friedman 1993; Chaudhuri and Behan 2000). In fact, it has been recently proposed that pathological fatigue could represent a perceptual disorder of the sensorimotor system, in which diminished attenuation of sensory inputs manifest as poor selective attention, leading to a high perceived effort even for simple daily living activities (Kuppuswamy 2017, 2023).

A difference between groups emerged also for sleep/fatigue and mood/apathy dimensions of the non-motor symptom scale. These data indirectly confirmed the effectiveness of safinamide on fatigue.

Other dimensions, such as the cardiovascular, urinary and sexual functions, were significantly different between responders and non-responders. This combination may suggest a “glutamatergic” cluster of non-motor symptoms. Further studies are needed to corroborate this observation.

Finally, we did not observe any improvement in UPDRS scores after treatment with safinamide, but we measured UPDRS at the peak of the levodopa dose and not at the end. Safinamide proved effective in reducing wearing-off and improving on time in PD patients with fluctuations (Schapira et al. 2017), which is probably the reason for this observation.

This study has several limitations. First, the study design, open-label and observational, did not include a control sample. Second, fatigue in our sample was evaluated using subjective scales. However, both FSS and PFS-16 obtained a “recommended” rating for screening and a “suggested” rating for severity when evaluated by an ad hoc task force of the Movement Disorder Society (Friedman et al. 2010)*.* We did not address anxiety, sleep disturbances and apathy with specific scales, but we adopted instead the NMSS which is a reliable, valid and precise instrument to assess non-motor symptoms in Parkinson’s disease (Martinez-Martin et al. 2009)*.*

Our sample of PD patients was already relatively small before it was divided into two groups; therefore, it may be difficult to generalize our findings to the whole population of PD patients who suffer from fatigue.

Our data confirm that fatigue is a symptom intrinsic to the pathological substrates associated with PD rather than secondary to motor impairment, mood disorders, or medications.

After 6 months of treatment with safinamide, fatigue improved in fluctuating PD. More than 40% of patients experienced the absence of fatigue at follow-up, indicating that drugs that interact with multiple neurotransmission systems, such as safinamide, could reduce this symptom and ameliorate the quality of life, possibly due to the fact that, rather than a reflection of a single neurotransmitter deficiency, the pathogenesis of PD-related fatigue is multifactorial and a dysfunction in the glutamatergic system may contribute to its development.

## Data Availability

Data are available from the corresponding author on reasonable request.
